# A Sneeze Away From Paralysis: A Unique Case of Spontaneous Spinal Intradural Hematoma

**DOI:** 10.7759/cureus.81738

**Published:** 2025-04-05

**Authors:** Sofia Vedor, Tiago Pedro, Miguel Peliteiro, Gonçalo Alves, Duarte Vieira

**Affiliations:** 1 Neuroradiology Department, Unidade Local de Saúde de São João, Porto, PRT

**Keywords:** conservative treatment, mielopathy, sneeze, spinal intradural hematoma, spontaneous

## Abstract

Spontaneous spinal intradural hematomas (SSIH) are exceptionally rare and typically associated with trauma, coagulopathy, or vascular malformations. Even rarer are cases triggered by seemingly benign events such as sneezing.

We report the case of a 72-year-old male who developed acute neurological symptoms following a sneezing episode despite having no prior history of trauma or coagulopathy. The patient presented with a band-like pain localized to the periumbilical region and caudal hypesthesia, persisting for one week.

Spinal MRI revealed an extensive intradural hematoma extending from the pre-pontine cistern to T12, predominantly along the ventral and dorsal aspects of the spinal cord. This hematoma caused significant cord compression and signs of myelopathy on the dorsal segment. Remarkably, the patient recovered full lower limb motor function through conservative management without requiring surgical intervention. However, intermittent self-catheterization remained necessary due to persistent urinary retention, likely multifactorial in origin.

This case highlights the importance of considering non-traumatic events such as sneezing as potential triggers for SSIH, even in the absence of classical risk factors, and underlines the value of individualized, imaging-guided management approaches. A literature review further highlights the rarity of such presentations and underscores the need for continued investigation into their underlying pathophysiology.

## Introduction

Spontaneous spinal intradural hematomas (SSIHs) are exceedingly rare and are most often associated with trauma, coagulopathy, or vascular malformations. Even less common are cases triggered by minor physiological events, such as coughing or sneezing, particularly in individuals without identifiable predisposing factors [1-3].

The underlying mechanism in such cases is believed to involve a sudden and significant increase in intrathoracic and intraspinal venous pressure during events like sneezing. This abrupt pressure surge may lead to the rupture of delicate radiculomedullary or bridging veins within the subdural or subarachnoid compartments of the spinal canal [4,5]. Clinically, SSIHs typically present with sudden-onset back or radicular pain followed by rapidly progressive neurological deficits. Delayed recognition can result in severe and potentially irreversible outcomes [1,2].

This report presents what we believe to be the first documented case of an SSIH precipitated by sneezing in an otherwise healthy individual.

## Case presentation

A 72-year-old male with no significant medical history nor regular medication use presented with a seven-day history of band-like pain localized to the periumbilical region, accompanied by caudal hypoesthesia. The clinical picture began immediately after a sneezing episode, with the patient experiencing sudden-onset, severe axial pain radiating along the entire spine. Over the subsequent four days, he reported progressive neurological decline, culminating in the abrupt onset of bilateral lower limb weakness and hypoesthesia. At admission to a secondary care facility, a neurological examination demonstrated paraparesis with Medical Research Council (MRC) grade 4 strength in both lower limbs and an inability to ambulate, with preserved deep tendon reflexes. Sensory examination confirmed caudal hypoesthesia. In addition, the patient reported acute urinary retention, which required bladder catheterization. He denied any preceding trauma, fever, systemic symptoms, or recent infection. No sensory deficits were noted in the upper limbs. Laboratory workup revealed normal platelet counts and standard coagulation parameters (prothrombin time {PT}, activated partial thromboplastin time {aPTT}), with mildly elevated D-dimer, C-reactive protein, and fibrinogen levels (Table 1). In the absence of clinical or microbiological evidence of infection, the mildly elevated acute-phase reactants were attributed to the inflammatory response secondary to the hematoma. Besides, no laboratory or imaging findings suggestive of an underlying neoplastic process were identified during hospitalization or follow-up.

**Table 1:** Summary of laboratory test results. D-dimer is a marker of increased fibrin turnover, while C-reactive protein and fibrinogen are acute-phase reactants that rise in response to inflammation and tissue injury. Collectively, these findings suggest activation of inflammatory and coagulation pathways. aPTT, activated partial thromboplastin time; INR, international normalized ratio; ALT, alanine transaminase; AST, aspartate transferase; GGTP, gamma-glutamyl transpeptidase, ESR, erythrocyte sedimentation rate.

Test	Reference range	Result
White blood cells	4.0-11.0 x 10^3^/μL	10.27
Erythrocytes	4.48 x 10^6^/μL	4.5 – 5.5
Hemoglobin	15.3 g/dL	13 – 17
Hematocrit	45.3%	40.0 – 50.0
Platelets	249 x 10^3^/μL	150.0 – 400.0
aPTT	30.0 s	24.2 – 36.4
Prothrombin time	11.9 s	11.0 – 12.8
INR	1.07	0.8 – 1.2
D-Dimer	740 ng/mL	< 500
Fibrinogen	5.7 g/L	2.0 – 4.0
Sodium	144 mmol/L	135-145
Potassium	4.88 mmol/L	3.50-5.00
Chloride	106 mmol/L	95-105
Urea	33 mg/dL	10 – 50
Creatinine	0.88 mg/dL	0.7 – 1.2
Uric acid	3.9 mg/dL	3.4 – 7.0
ALT	14 U/L	10 – 44
AST	14 U/L	10 – 34
Total bilirubin	0.51 mg/dL	0.20 – 1.00
GGTP	30 U/L	10 – 66
Albumin	4.64 g/dL	3.4 – 4.8
C-reactive protein	2.79 mg/dL	< 0.5
ESR	10 mm	0 – 30

**Figure 1:**
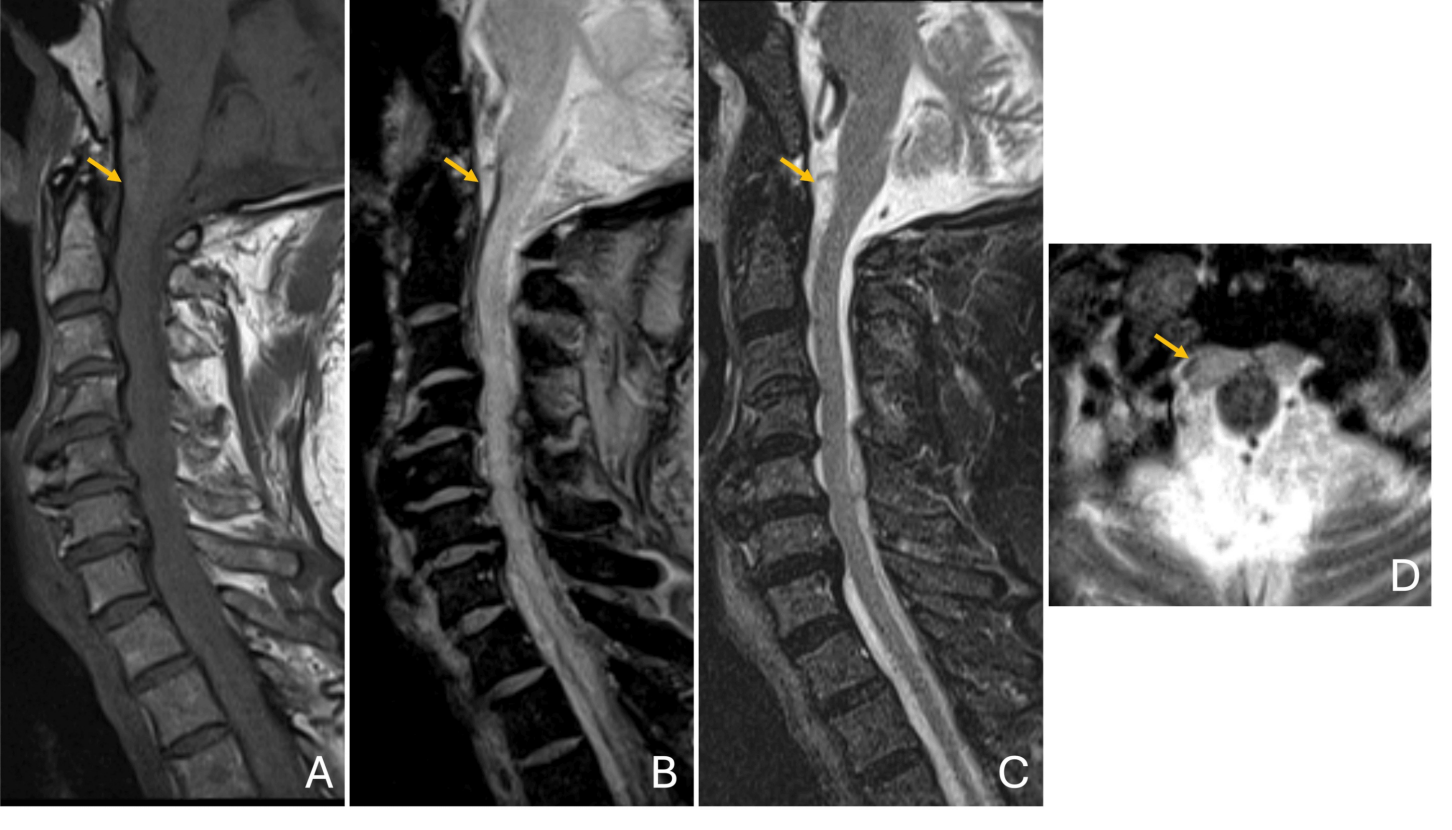
Cervical MRI. Large hematoma extending from the pre-pontine cistern, predominantly anterior to the spinal cord, with involvement throughout the entire cervical segment and the upper thoracic region, partially captured within the field of view. The hematoma exhibited isointense signal on T1-weighted imaging (A), blooming on T2* (B), and hyperintensity on STIR (C), blending with the CSF signal but demonstrating hypointense signal on T2, particularly evident at the level of the cisterna magna (D, yellow arrow). MRI, magnetic resonance imaging; CSF, cerebrospinal fluid; STIR, short tau inversion recovery.

**Figure 2:**
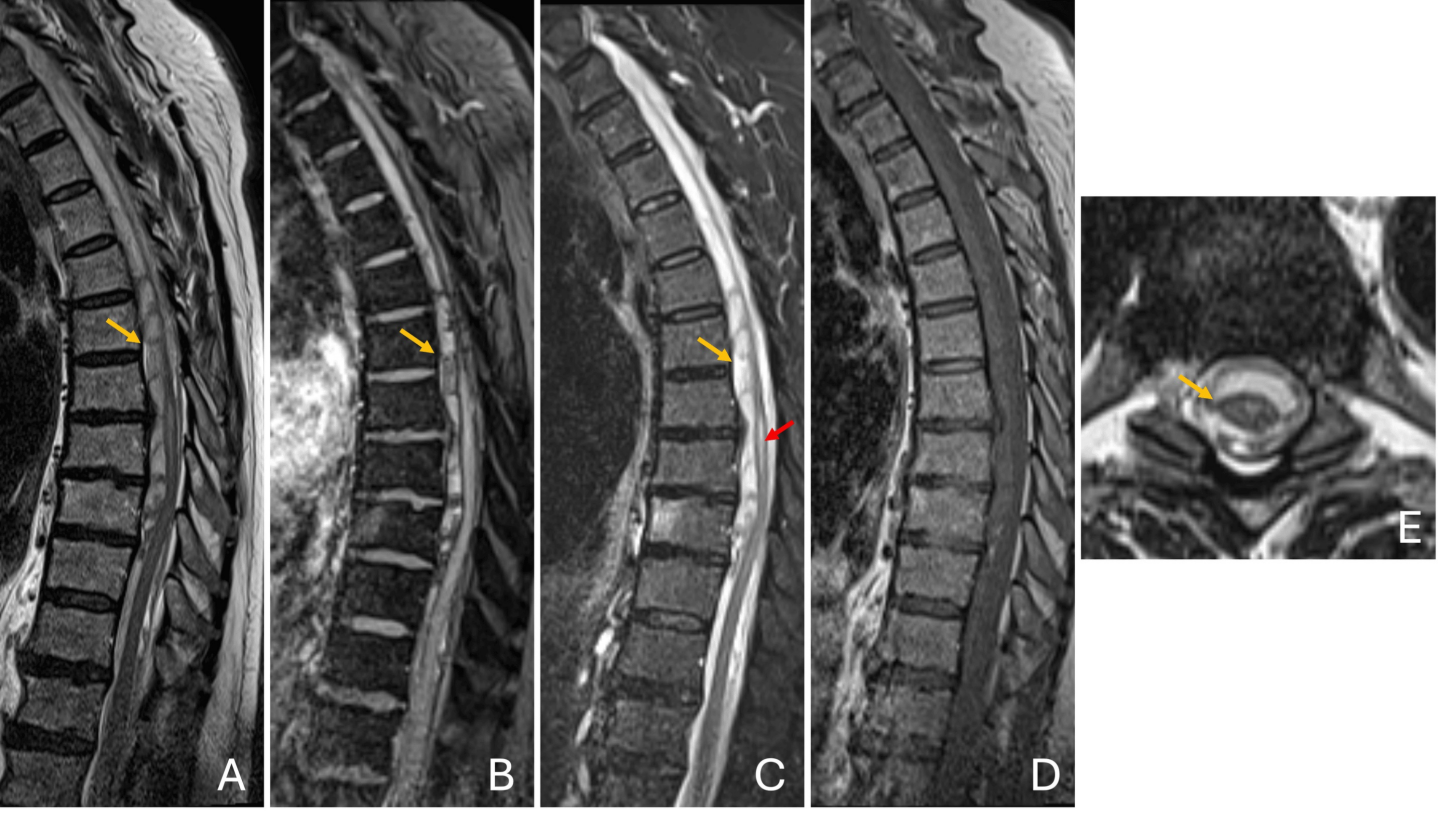
Dorsal MRI. Maximal thickness and septations were observed between T6 and T12 (yellow arrow), as demonstrated on T1-weighted imaging (A), T2* (B), and STIR (C), reaching 8 mm at the level of T8. The hematoma extended caudally to T12, causing posterior displacement and compression of the spinal cord, with centromedullary hyperintensity from T7 to T12, suggestive of edema (C, red arrow). While predominantly anterior, it also extended posteriorly and laterally, without evident restriction by the denticulate ligaments, supporting a subarachnoid location (E), though a subdural component could not be excluded. No abnormal contrast enhancement was observed on sagittal T1-weighted imaging, disfavoring the presence of vascular anomalies, namely arteriovenous shunts and cavernous malformations (D). MRI, magnetic resonance imaging; STIR, short tau inversion recovery.

Spinal magnetic resonance imaging (MRI) demonstrated a large intradural hematoma extending from the pre-pontine cistern to T12 (Figures 1, 2), predominantly anterior to the spinal cord, with maximal thickness and septations observed between T6 and T12, reaching 8 mm at T8 (Figure 2A-2D). The hematoma caused posterior displacement and compression of the spinal cord, with centromedullary areas of hyperintensity extending from T7 to T12, suggestive of edema (Figure 2C, yellow arrow). It also exhibited posterior and lateral distribution across all described levels, albeit in a smaller volume than its anterior component, without apparent limitation by the denticulate ligaments. This distribution favored a subarachnoid location of the hematic content, though coexisting subdural hemorrhage couldn’t be entirely excluded. No epidural hematoma or hyperintense cord signal abnormalities were observed in the cervical segment. The absence of enhancement on post-contrast sequences disfavored underlying vascular anomalies, namely arteriovenous shunts and cavernous malformations (Figure 2D).

**Figure 3:**
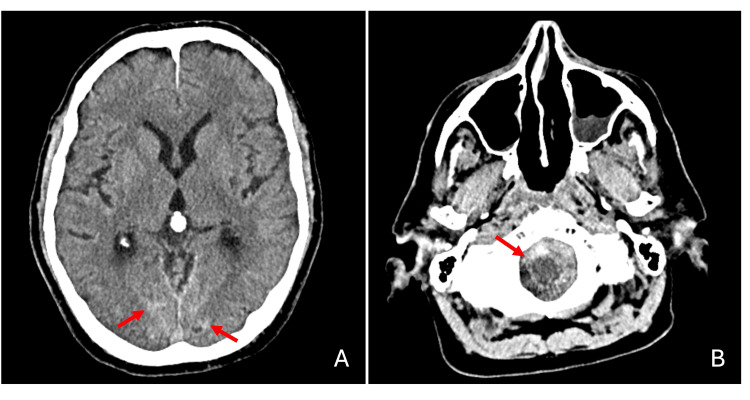
Brain CT. Mild subarachnoid hemorrhage within the posterior occipital sulci (A, red arrow), with a more significant distribution in the cisterna magna, predominantly along its anterior and left lateral aspects (B, red arrow), as previously documented on cervical MRI. CT, computed tomography; MRI, magnetic resonance imaging.

A complementary brain CT revealed mild subarachnoid hemorrhage within the posterior occipital sulci, superior cerebellar folia, and basal cisterns (Figure 3), likely due to redistribution of the hematic content, with no evidence of hydrocephalus. 

During hospitalization, a spinal CT angiography (CTA) and spinal digital subtraction angiography (SpDSA) were also performed for the unequivocal exclusion of dural arteriovenous fistulas, both of which were negative. The patient demonstrated gradual neurological improvement with conservative management, including analgesia and supportive care. Follow-up imaging three weeks after admission showed partial resorption of the intradural hematoma, with caudal redistribution along the posterior aspect of the thecal sac from L3 to S1, leading to a progressive reduction in the regional mass effect.

After the initial three months of hospitalization, the patient was transferred to a private rehabilitation facility to continue multidisciplinary care. At that time, he had already regained full motor strength in the lower limbs (grade 5 bilaterally), avoiding the need for surgical intervention. During hospitalization, arterial hypertension was newly diagnosed, and antihypertensive therapy was initiated and maintained long-term. He was discharged definitively after a total of four and a half months of hospitalization, with persistent vesico-sphincter dysfunction requiring intermittent self-catheterization. Non-complicated grade 2 pressure ulcers over both heels were also present, attributed to prolonged immobility.

At both the six-month and one-year evaluations, the patient remained on intermittent self-catheterization without requiring nighttime drainage. Given the pre-existing lower urinary tract symptoms, namely nocturia and incomplete bladder emptying, with subsequent ultrasound diagnosis of benign prostatic hyperplasia, urinary retention was considered multifactorial. Tamsulosin was initiated during hospitalization and continued throughout follow-up. The patient also reported occasional mild lower back pain upon awakening, attributed to poor nocturnal positioning and without associated red-flag symptoms. Although the patient did not attend formal outpatient physiotherapy, he maintained daily short walks and mobility exercises, which contributed to preserving functional autonomy.

## Discussion

SSIH is an exceptionally rare condition, most commonly associated with trauma, coagulopathy, and vascular malformations [1-3]. While a few cases of epidural hematomas triggered by sneezing have been described [4,5], no documented SSIH directly linked to this mechanism exists in the literature, making this a uniquely reported case.

Sneezing induces a sudden and transient increase in intrathoracic and intraspinal venous pressure, potentially leading to the rupture of fragile epidural vessels, resulting in hematoma formation [4,5]. However, the pathophysiology of SSIH remains incompletely understood, particularly in cases without identifiable risk factors, making it a diagnosis of exclusion [3,6-9]. The most plausible explanation for SSIH involves the rupture of radiculomedullary veins and the rich capillary network within the intradural space, given their valveless nature and vulnerability to sudden pressure changes. Minor trauma or transient increases in intrathoracic pressure - such as those induced by physical exertion or sneezing - can elevate spinal venous pressure. If cerebrospinal fluid (CSF) pressure fails to counterbalance this force, vessel rupture may occur, leading to hemorrhage within the subarachnoid or subdural spaces. Given the anatomical continuity between these compartments, distinguishing the exact origin of the hemorrhage can be challenging, and a mixed bleeding pattern is often observed [3,7,8]. Consequently, the term “intradural extramedullary hematoma” was introduced to encompass these presentations more accurately [9]. Arterial sources, while not entirely excluded, are less likely in the absence of active bleeding or vascular anomalies on imaging. Besides, spinal subarachnoid hemorrhage rarely presents as a hematoma, owing to the diluting and redistributing effect of the CSF, unless the hematoma is sufficiently large to block the CSF flow [7].

Most spinal hematomas are typically located dorsally to the spinal cord, predominantly in the cervicothoracic and thoracolumbar regions. In contrast, subarachnoid hematomas can extend extensively along the entire subarachnoid space [2]. The typical clinical presentation of SSIH includes acute back pain, often associated with sensory disturbances, paralysis, and sphincter dysfunction, features that were also observed in our patient.

MRI remains the gold standard for this diagnosis, as it provides detailed visualization of the hematoma and its relationship to the spinal cord [1-10]. In this case, it revealed an extensive hematoma extending from the pre-pontine cistern to T12, with significant anterior compression of the spinal cord and myelopathy. The lack of gadolinium enhancement and the absence of vascular abnormalities further supported a venous origin. CT provided additional insights, revealing redistribution of hematic content into the ventricles and subarachnoid spaces.

The management of SSIH depends on the severity of neurological deficits and the progression of symptoms. Surgical decompression is typically reserved for cases with rapid neurological deterioration or severe deficits. In contrast, conservative management may be appropriate in stable patients, as the spontaneous resolution of hematomas has been documented [3,10]. This patient experienced gradual neurological improvement with conservative treatment, ultimately achieving a full recovery, underscoring the potential for non-surgical management in selected cases. Lastly, although this is a single case, the one-year follow-up reinforces the notion of a sustained neurological recovery.

A review of the literature highlights the lack of SSIH triggered by sneezing. This case adds to the limited body of evidence suggesting that sneezing alone can precipitate significant spinal pathology. It also underscores the importance of early imaging in patients with acute neurological deficits, as timely diagnosis and appropriate management can lead to favorable outcomes.

## Conclusions

This case demonstrates the importance of recognizing non-traumatic triggers, such as sneezing, as potential causes of SSIH. The absence of traditional risk factors should not preclude clinicians from considering this diagnosis in patients with acute neurological deficits.

Imaging modalities, particularly MRI, played a pivotal role in identifying the extent and characteristics of the hematoma, allowing for appropriate management without surgical intervention. The favorable outcome highlights the importance of individualized treatment plans based on clinical presentation and imaging findings. A multidisciplinary approach, combining timely imaging and clinical expertise, remains essential for optimizing patient outcomes in such complex and uncommon presentations.
